# Peripheral vasoconstriction is not elevated during hyperreactive responses to the cold pressor test: a cross-sectional study

**DOI:** 10.3389/fphys.2025.1532992

**Published:** 2025-03-12

**Authors:** Jon Stavres, Anabelle Vallecillo-Bustos, Sarah Parnell, Ryan S. Aultman, Ta’Quoris A. Newsome, Sydney H. Swafford, Abby T. Compton, Rhett C. Schimpf, Sophia N. Schmidt, Carstell Lee, Austin J. Graybeal

**Affiliations:** ^1^ School of Kinesiology and Nutrition, The University of Southern Mississippi, Hattiesburg, MS, United States; ^2^ School of Health Sciences, Kent State University, Kent, OH, United States; ^3^ School of Medicine, University of Mississippi Medical Center, Jackson, MS, United States

**Keywords:** vascular conductance, blood flow, cold stress, sympathetic, handgrip

## Abstract

**Background:**

Individuals demonstrating increases in systolic systolic blood pressure or diastolic diastolic blood pressure blood pressure of at least 15 mmHg are considered hyperreactors to the cold pressor test (CPT). However, it remains unclear if peripheral vasoconstriction is similarly exaggerated during the CPT in these individuals.

**Methods:**

Fifty-five individuals (54.5% non-White, 67.3% female) performed a single-visit study including a 2-min CPT of the foot, a 2-min bout of rhythmic handgrip exercise ([HG] 25% maximal voluntary contraction), and a 2-min combined trial (CPT + HG). Beat-by-beat heart rate (HR), blood pressure, and forearm blood flow (FBF) were continuously recorded, and vascular conductance (FVC) was calculated as FBF/mean arterial pressure (MAP).

**Results:**

Hyperreactors (*n* = 21) demonstrated exaggerated increases in blood pressure and rate pressure product during the CPT compared to normoreactors (*n* = 34; all *p* < 0.001), while no significant differences were observed for ΔFBF (*f* = 1.33, *p* = 0.259) or ΔFVC responses (*f* = 2.10, *p* = 0.083). Results also indicated a blunted increase in ΔMAP during the CPT + HG trial compared to the CPT only trial in hyperreactors (*f* = 6.95, *p* < 0.001), which was not observed in normoreactors (*f* = 0.982, *p* = 0.420), and a blunted ΔFVC response during the CPT + HG trial in hyperreactors compared to normoreactors (*f* = 2.57, *p* = 0.039). When analyzed separately, the blood pressure responses to HG exercise were also significantly exaggerated in hyperreactors compared to normoreactors (all *p* < 0.001), while ΔFBF and ΔFVC responses were not (both *p* ≥ 0.701).

**Conclusion:**

These findings indicate that hyperreactive blood pressure responses to the CPT are not accompanied by increased peripheral vasoconstriction. Moreover, handgrip exercise attenuates hyperreactive blood pressure responses to the CPT.

## Introduction

The cold-pressor test (CPT) is a commonly used assessment of sympathetic reactivity in humans. Originally described by Hines and colleagues in 1936 (Hines and Brown, 1936), the CPT elicits robust increases blood pressure and heart rate (HR) in response to acute (generally 2-min) bouts of cold-water (∼4°C) immersion of the hand or foot. Notably, the magnitude of blood pressure increases observed during the CPT has been associated with poor cardiovascular health, particularly in individuals classified as hyperreactors (defined as an increase in systolic [SBP] or diastolic blood pressure [DBP] of ≥15 mmHg; (Wood et al., 1984; Kasagi et al., 1995; Han et al., 2022)). Specifically, Wood et al. (1984) reported that 71% of individuals classified as CPT hyperreactors in a single cohort developed future hypertension compared to only 19% of individuals classified as normoreactors in the same cohort. These results were further supported by Kasagi et al. (1995), who reported that individuals with a hyperreactive systolic blood pressure (SBP) response to the CPT presented with a higher relative risk of future hypertension. Accordingly, understanding the mechanisms contributing to the hyperreactive response to the CPT can inform both the etiology of increased cardiovascular disease risk and potential treatment options for individuals classified as high risk. Unfortunately, with the exception of prior evidence of exaggerated increases in pulse-wave augmentation index in hyperreactors (Moriyama and Ifuku, 2010), there is limited data available focused on the hemodynamic factors contributing to this hyperreactive response.

In healthy adults, the increases in HR and blood pressure observed during the CPT are driven by concomitant increases in cardiac and muscle sympathetic nerve activity [MSNA; (Victor et al., 1987)], leading to decreases in blood flow and vascular conductance of the peripheral limbs (Stephens et al., 2023; Stavres et al., 2024). However, the timeline of these changes occur out of sequence, with HR increasing most during the first 30 s of the test and MSNA demonstrating a delayed response (Victor et al., 1987; Yamamoto et al., 1992). Likewise, prior evidence indicates that individuals with obesity, who are known to have elevated resting MSNA (Alvarez et al., 2002), experience larger increases in MSNA during the CPT compared to individuals without obesity, despite no differences in blood pressure responses (Park et al., 2012). Similarly, prior research also indicates that individuals with metabolic syndrome demonstrate elevated MSNA and blood pressure at rest (Grassi et al., 2005; Limberg et al., 2012), but do not demonstrate exaggerated blood pressure responses to the CPT (Stavres et al., 2024). Taken together, these findings indicate that 1) central and peripheral hemodynamic responses likely contribute independently to the exaggerated blood pressure responses observed in individuals who are hyperreactive to the CPT, and 2) increases in MSNA alone do not guarantee a greater contribution of peripheral vasoconstriction to these hyperreactive responses.

With this in mind, this study aimed to determine if peripheral vasoconstriction is similarly exaggerated during a CPT in individuals classified as CPT hyperreactors. Based on the robust increases in MSNA during the later stages of the CPT in healthy adults (Victor et al., 1987), during which blood pressure tends to increase most, we hypothesized that peripheral hemodynamics, represented as the relative changes in forearm blood flow (ΔFBF) and forearm vascular conductance (ΔFVC), would decrease more during the CPT (via cold-water foot immersion) in individuals classified as hyperreactors compared to individuals classified as normoreactors. Secondarily, we also hypothesized that exaggerated peripheral vasoconstriction during the CPT in hyperreactors would oppose the vasodilatory response observed during periods of voluntary muscle activation, indicated by blunted increases in FBF and FVC during combined rhythmic handgrip exercise (HG) and CPT.

## Methods

### Participants and study design

**FIGURE 1:**
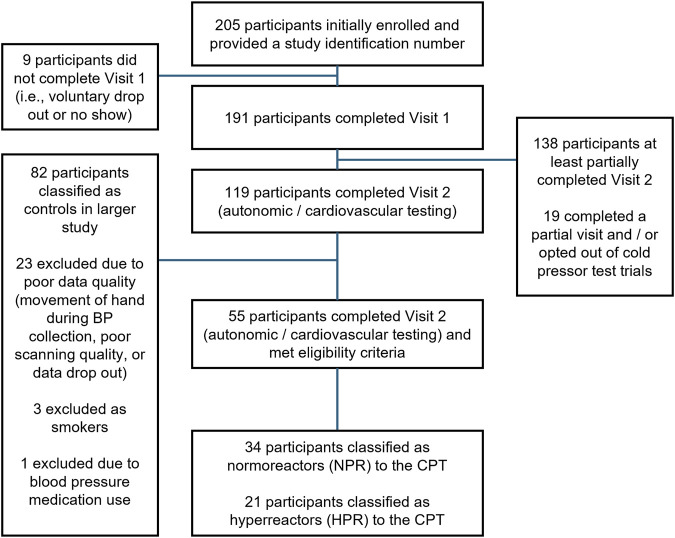
CONSORT diagram. CPT, cold pressor test.

The data for this study was collected as part of a larger investigation examining the use of a continuous index of metabolic syndrome severity for detecting early autonomic and cardiovascular dysfunction in young adults (NCT05885672). To be included in this analysis, participants must have been classified as a control participant (not meeting the diagnostic criteria for metabolic syndrome), be free of any known cardiovascular, metabolic, or renal diseases, must not be an active smoker (≥6 months), and must not be prescribed or taking blood pressure medications. Participants were also excluded if they did not perform the CPT portion of the larger study, or if they demonstrated a hypotensive response to the CPT or HG (defined as a change in mean arterial pressure [MAP] of <0 mmHg across the entire CPT period). Participants were recruited via word of mouth and distribution of study advertisements in the Hattiesburg, MS region, and a breakdown of the enrollment period is provided as a CONSORT diagram in Figure 1. Of note, assessments of lower leg and upper arm reactive hyperemia were collected prior to the CPT, HG, and combined CPT + HG assessment for all participants (data not included in this analysis), and ≥10 min of washout separated all experimental trials.

Data for this study was collected across two visits. The first visit included a cardiometabolic prescreening, and all cardiovascular responses were assessed in the second visit. A total of 191 individuals originally participated in the first visit of this study, 138 of whom at least partially completed the second visit (visits described below). Of these individuals, fifty-five met the criteria defined above, thirty-four of whom were classified as CPT hyperreactors (HPR group; increase in SBP, DBP, or MAP of ≥15 mmHg), and twenty-one of whom were classified as normoreactors (NPR group; increases in SBP, DBP, or MAP of <15 mmHg). The overall sample (*n* = 55) was predominantly female (*n* = 37), and was 41.8% non-Hispanic White (*n* = 23), 3.7% Hispanic White (*n* = 2), 20% non-Hispanic Black/African American (*n* = 11), 1.8% Hispanic Black/African American (*n* = 1), 30.9% non-Hispanic Asian (*n* = 17), and 1.8% Hispanic Native American (*n* = 1). The mean age of the sample was 21 ± 2 years, with a BMI of 23.9 ± 3.5 kg/m^2^, SBP of 111 ± 11 mmHg, DBP of 75 ± 10 mmHg, and a RHR of 67 ± 10 bpm. Of the thirty-seven females included in the final analysis, the start date of the most recent menstrual cycle was recorded in twenty participants, with autonomic testing occurring an average of 17 ± 10 days from the start of their most recent menstrual cycle. All other demographics and group comparisons are presented in Tables 1, 2. All participants provided written informed consent, and all protocols were approved by the local ethics committee (IRB# 23-0446).

**TABLE 1:** Participant demographics.

	Whole sample	NPR	HPR	*t*	*p*
*n*	55	34	21	—	—
Male (*n*)	18	9	9	—	—
Female (*n*)	37	25	12	—	—
Race					
White (*n*)	25	17	8	—	—
BAA (*n*)	12	6	6	—	—
Asian (*n*)	17	11	6	—	—
NA (*n*)	1	0	1	—	—
Baseline Characteristics					
Age (*yrs*)	21 ± 2	21 ± 2	22 ± 3	−1.49	0.142
Height (*cm*)	166.5 ± 8.4	166.4 ± 7.9	166.7 ± 9.3	−0.17	0.870
Weight (*kg*)	66.4 ± 11.6	66.5 ± 10.9	66.2 ± 12.9	0.09	0.925
BMI (*kg/m* ^*2*^)	23.9 ± 3.5	24.0 ± 2.9	23.8 ± 4.4	0.12	0.901
MVC (*kg*)	34.5 ± 10.3	33.2 ± 8.4	36.6 ± 12.8	−1.17	0.245
RSBP (*mmHg*)	111 ± 11	110 ± 10	112 ± 13	−0.69	0.495
RDBP (*mmHg*)	75 ± 10	74 ± 10	77 ± 8	−1.28	0.205
RHR (*bpm*)	67 ± 10	68 ± 9	65 ± 10	1.06	0.295
TC (*mg/dL*)	156 ± 31	158 ± 27	152 ± 37	0.64	0.526
HDL-C (*mg/dL*)	52 ± 12	51 ± 12	53 ± 12	−0.71	0.479
LDL-C (*mg/dL*)	87 ± 31	88 ± 27	86 ± 40	0.19	0.847
FBG (*mg/dL*)	87 ± 5	87 ± 5	86 ± 6	0.24	0.810
BF% (*%*)	30.0 ± 9.7	31.5 ± 9.6	27.5 ± 9.5	1.53	0.132
FM (*kg*)	19.3 ± 8.3	20.4 ± 8.4	17.6 ± 8.0	1.24	0.222
cBRS_all_ (*mmHg/ms*)	22.2 ± 12.7	23.2 ± 14.3	20.5 ± 9.6	0.78	0.441
cBRS_up_ (*mmHg/ms*)	28.4 ± 18.3	29.6 ± 20.0	26.6 ± 15.6	0.59	0.559
cBRS_down_ (*mmHg/ms*)	17.3 ± 9.0	17.6 ± 10.0	16.9 ± 7.4	0.31	0.761
RHBF-TTP (*sec*)	10.7 ± 20.6	8.4 ± 4.2	14.4 ± 33.1	−1.05	0.297

NPR, normoreactor group; HPR, hyperreactor group; BAA, Black/African American; NA, Native American/Alaskan Native; BMI, body mass index; MVC, maximal voluntary contraction; RSBP, resting systolic blood pressure; RDBP, resting diastolic blood pressure; RHR, resting heart rate; TC, total cholesterol; HDL-C, high-density lipoprotein cholesterol; LDL-C, low-density lipoprotein cholesterol; FBG, fasting blood glucose; BF%, body fat percentage; FM, total fat mass; cBRS_all_, spontaneous cardiovagal baroreflex gain of all identified sequences; cBRS_up_, baroreflex gain of all up-ramping sequences; cBRS_down_, baroreflex gain of all down-ramping sequences; RHBF-TTP, time to peak blood flow during brachial reactive hyperemia trial.

**TABLE 2:** Baseline comparisons across race and sex.

	Sex				Race					
	Male	Female	*t*	*p*	White	BAA	Asian	NAA	*f* ^*a*^	*p*
*n*										
Male (*n*)	18	0	—	—	5	3	10	0	—	—
Female (*n*)	0	37	—	—	20	9	7	1	—	—
Race									—	—
White (*n*)	5	20	—	—	—	—	—	—	—	—
BAA (*n*)	3	9	—	—	—	—	—	—	—	—
Asian (*n*)	10	7	—	—	—	—	—	—	—	—
NA (*n*)	0	1	—	—	—	—	—	—	—	—
Baseline Characteristics										
Age (*yrs*)	21 ± 3	21 ± 2	0.72	0.474	21 ± 2	22 ± 2	21 ± 3	22	1.54	0.234
Height (*cm*)	173.0 ± 6.5	163.3 ± 7.3	−4.79	<0.001	167.7 ± 7.7	166.8 ± 10.5	164.7 ± 8.2	163.5	0.67	0.519
Weight (*kg*)	68.3 ± 12.8	65.4 ± 11.0	−0.87	0.388	69.2 ± 11.1	72.9 ± 12.7	58.1 ± 6.3^b,c^	58.9	12.2	<0.001
BMI (*kg/m* ^*2*^)	22.7 ± 3.3	24.5 ± 3.5	1.81	0.076	24.5 ± 2.7	26.3 ± 4.3	21.5 ± 2.5^b,c^	22.0	9.38	<0.001
MVC (*kg*)	43.5 ± 11.5	30.1 ± 6.1	−5.69	<0.001	34.3 ± 9.2	39.0 ± 14.2	32.1 ± 8.2	26.5	1.15	0.333

BAA, Black/African American; NAA, Native American/Alaskan Native; BMI, body mass index; MVC, maximal voluntary contraction; RSBP, resting systolic blood pressure; RDBP, resting diastolic blood pressure; RHR, resting heart rate; TC, total cholesterol; HDL-C, high-density lipoprotein Cholesterol; LDL-C, low-density lipoprotein cholesterol; FBG, fasting blood glucose; BF%, body fat percentage; FM, total fat mass.

^a^ Native American participant (*n* = 1) was removed from ANOVA, comparisons.

^b^ Significantly different from White participants.

^c^ Significantly different from Black/African American participants. Significance accepted at *p* < 0.050.

### Cardiometabolic prescreening

Visit 1 served as a cardiometabolic prescreening. Participants arrived at the first visit at least 8 hours postprandial, including caffeine, and having abstained from alcohol and over-the-counter medications for 12 hours and moderate-to-vigorous exercise for 24 hours prior to their visit. Upon arrival, waist circumference (WC) was collected at the level of the iliac crest using a spring-loaded tape measure according to published recommendations (Grundy et al., 2005), and height and weight were collected using a calibrated scale and stadiometer. Next, approximately 40 µL of blood were collected via capillary fingerstick and analyzed for fasting blood glucose (FBG), low-density lipoprotein cholesterol (LDL-C), high-density lipoprotein cholesterol (HDL-C), total cholesterol (TC), and triglycerides (TRG) via a point-of-care lipid analyzer (Cholestech LDX, Abott, Abbott Park, IL). The lipid analyzer was calibrated upon opening each new batch of cassettes according to manufacturer recommendations using both high and low multianalyte control solutions, and the analyzers optical scanner was calibrated each day of testing using a standardized optical control cassette. Body composition was also collected as a descriptor variable via dual-energy x-ray absorptiometry (DEXA; GE Lunar idxa, GE Healthcare, Chicago, IL). For the larger investigation, these data were used to stratify individuals into two primary groups: individuals who met the National Cholesterol Education Panel’s ATP III criteria for metabolic syndrome, and control participants who did not meet these criteria. As noted previously, only individuals classified as control participants were included in this analysis.

### Cardiovascular assessments

Visit 2 consisted of a series of cardiovascular assessments designed to test the hemodynamic responses to cold-pressor activation and handgrip exercise. Participants arrived at this visit at least 8 hours postprandial and having abstained from over-the-counter medications for 12 hours and moderate-to-vigorous exercise for 24 hours prior. Upon arrival at this visit, participants were instrumented with a beat-by-beat finger blood pressure monitor (Finapres NOVA and Finapres NANO, Finapres Medical Systems, Enschede, The Netherlands) and a one-lead (Lead I) electrocardiogram, both of which were streamed into a multi-channel data acquisition system (PowerLab 8/35, AD Instruments, Colorado Springs, CO). The beat-by-beat blood pressure signal was calibrated to the brachial blood pressure value collected on the ipsilateral upper arm prior to the baseline period of each trial. After instrumentation, participants underwent a series of resting cardiovascular and autonomic function tests, including resting assessments of heart rate variability and spontaneous cardiovagal baroreflex sensitivity (cBRS), as well as assessments of post-occlusive reactive hyperemia in the lower leg and forearm. Of note, these experiments were collected to address a separate hypothesis, and therefore some (resting HRV and lower leg reactive hyperemia responses) are not discussed in this manuscript. However, cBRS and forearm reactive hyperemia are both reported as a baseline comparison between groups.

To evaluate cBRS, beat-by-beat measurements of SBP and r-r interval were extracted from a 10-min baseline period during which participants breathed to a metronome at seven breaths per minute. This breathing frequency is intended to control the influence of respiration on resting autonomic tone (Bernardi et al., 2001). From these data, the baroreflex gain of all up-ramping spontaneous blood pressure sequences (cBRS_up_), all down-ramping baroreflex sequences (cBRS_down_), and all combined sequences (cBRS_all_) were evaluated using the sequence method (Dias et al., 2016). The parameters for this method included a minimum change in SBP of at least 1 mmHg, a minimum change in cardiac interval of at least 5 ms, a minimum sequence length of at least three beats, an inter-beat belay of one beat, and a minimum r-value of 0.8.

For assessment of forearm reactive hyperemia, brachial blood flow velocity was continuously recorded from the left brachial artery via duplex Doppler ultrasound (GE Logiq P5, GE Healthcare, Chicago IL) using a linear array transducer (11 L) operating at 3.5 mHz throughout a 2-min baseline, followed by a 5-min period of forearm occlusion (occlusion confirmed as the loss of Doppler ultrasound pulse wave signal), and a 2-min period of reactive hyperemia. The time-to-peak blood flow velocity following the release of the cuff (RHBF-TTP) was recorded as an index of microvascular reactivity and included as a baseline comparison between groups. Following the assessment of post-occlusive reactive hyperemia, participants rested for a ∼10-min washout period before beginning the experiments involved in this investigation.

To test the hypothesis that individuals classified as hyperreactors to the CPT would demonstrate exaggerated peripheral vasoconstriction relative to individuals classified as normoreactors, all participants completed a 2-min cold pressor test of the left foot while beat-by-beat blood pressure and heart rate were continuously recorded. Of note, the protocol originally included a 3-min CPT period, however, this was reduced to 2-min following pilot testing after it was observed that 2-min achieved a robust blood pressure response without undue participant burden. Blood pressure data included mean arterial pressure (MAP; calculated as the average of all data points within each individual cardiac cycle), systolic blood pressure (SBP; identified as the peak pressure value within each cardiac cycle), and diastolic blood pressure (DBP; identified as the lowest pressure value within each cardiac cycle). Rate pressure produce (RPP) was also calculated as SBP * HR for each heartbeat and is reported as an index of myocardial oxygen demand. Likewise, brachial blood flow velocity was also continuously recorded from the ipsilateral (left) brachial artery as described above, and the calculations for forearm blood flow FBF and FVC are included in the following sections. Water temperature was not directly controlled in this trial, but water temperature was recorded in a subset of trials (*n* = 3) and was maintained between 3° and 4°C.

To test the secondary hypothesis that exaggerated sympathetic vasoconstrictor responses to the CPT would oppose exercise induced vasodilatory responses in individuals classified as hyperreactors, all participants also performed a 2-min bout of rhythmic handgrip exercise (HG), followed by a 2-min bout of combined CPT + HG. All three trials (CPT, HG, and CPT + HG) were separated by a 10-min rest period. To control for relative intensity, maximal voluntary contractions (MVC) were evaluated in triplicate at baseline, and HG intensity was assigned as 25% of the predetermined MVC. MVC testing, and the subsequent HG exercise, was performed using an analog handgrip dynamometer fitted with a potentiometer, which supplied real-time force output to the multi-channel data acquisition system (PowerLab, AD Instruments, Colorado Springs, CO) at 1 kHz sampling frequency. The force output signal was calibrated to the analog force recording using a two-point calibration procedure (20 kg and 40 kg) prior to the collection of MVCs. All HG exercise was performed using a 1:1 work to rest ratio at 60 contractions per minute, maintaining a light-to-moderate intensity that promoted quality Doppler imaging during active contraction. Like the CPT, beat-by-beat brachial blood flow velocity, HR, and blood pressure were continuously recorded during both the HG and CPT + HG trials. In addition to serving as an index of vasoconstrictor responses during the CPT, the selection of the brachial scanning location also provided direct assessments of the hyperemic responses to HG exercise.

### Near-infrared spectroscopy (NIRS)

In addition to the collection of beat-by-beat changes in central hemodynamic responses, skeletal muscle oxygenation was of the anterior forearm muscles was collected via continuous-wave NIRS (PortaLite, Artinis Medical Systems, Elst, Netherlands). This system provides measurements of oxygenated hemoglobin saturation (O_2_Hb), deoxygenated hemoglobin saturation (DO_2_Hb), total hemoglobin saturation (tHb), and tissue saturation index (TSI). Data were collected at 10 Hz frequency throughout the experimental trials, and down sampled to 1 Hz prior to analysis. These data are each reported as a mean change from the baseline timepoint (described below), with the exception of TSI, which is reported as the mean value (%) across all time points. Of note, NIRS data were collected in this study as a secondary marker of the microvascular responses to CPT and HG, with the expectation that individuals in the HPR group would demonstrate greater decreases in tHb and O_2_Hb during the CPT compared to the NPR group if peripheral vasoconstriction is exaggerated in the HPR group.

### Data computation

Each trial began with a 2-min baseline, followed by a 2-min experimental trial. MAP, SBP, DBP, and HR were recorded across each individual cardiac cycle using a custom macro-enabled r-wave detection program (accuracy was cross-checked by an investigator) and averaged across the final 30-s of baseline, and each 30-s increment during the 2-min CPT, resulting in five total timepoints of interest (baseline [BL], and thirty- [30p], sixty- [60p], ninety- [90p], and 120 s [120p] post immersion). FBF was calculated as the product of mean flow velocity * (π * ((mean diameter/2) ^2^)) * 60, and was also reported across each 30-s bin. Brachial diameter was collected in triplicate at the t-wave of three separate cardiac cycles within each 30-s bin and averaged to provide a single diameter value for each timepoint. FVC was then calculated as the quotient of FBF/MAP for each timepoint. Both FBF and FVC were used as indices of the global vasoconstrictor response to the CPT, as well as the opposed (HG + CPT) and unopposed (HG only) hyperemic responses to HG exercise. As noted above, NIRS data were collected at 10 Hz and down sampled to 1 Hz before being averaged across the same timepoints (BL, 30p, 60p, 90p, and 120p).

### Statistical analyses

Based on an expected moderate effect size of η_p_^2^ = 0.06, a power analysis (conducted using G*Power version 3.1.9.7 software (Faul et al., 2007)) indicated that at least twenty participants (ten per group) would be required to achieve statistical significant group (two groups) by time interactions (five timepoints; significance accepted at *p* < 0.05) with a desired power of 0.80. However, as noted previously, data for this study was collected as part of a larger investigation, and therefore all eligible control participants were included in this analysis, thus reducing the risk of selection bias. After collecting all data, participant demographics were compared between the NPR and HPR groups using independent samples *t*-tests. Likewise, a combination of *t*-tests and one-way analyses of variance (ANOVA) were used to test for baseline differences between the HPR vs NPR groups, between males and females, and across racial groups. Next, multifactorial repeated measures analyses of variance (RMANOVA) were used to test for group (HPR vs NPR) by time (BL, 30p, 60p, 90p, and 120p) interactions for all variables within the CPT trial independently. This tested the main hypothesis that peripheral vasoconstriction responses would also be exaggerated in the HPR group during the CPT. Next, three-way RMANOVA were used to test for group by condition (CPT vs CPT + HG) by time (BL, 30p, 60p, 90p, 120p) interactions for all measured variables. This was intended to determine if HG exercise and CPT responses interacted differently between the HPR and NPR groups. Lastly, the HG trials were also examined independently using separate two-group (HPR vs NPR) by five timepoint (BL, 30p, 60p, 90p, and 120p) RMANOVA. These analyses specifically addressed independent questions regarding group differences in the HG responses. The primary outcome measures for all analyses included ΔFBF, ΔFVC (both as markers of peripheral vasoconstriction), ΔMAP, ΔSBP, ΔDBP, and ΔHR (as markers of the hyperreactive CPT response), and the secondary outcome measures included cBRS, RHBF-TTP, ΔRPP, TSI, ΔO_2_Hb, ΔDO_2_Hb, and ΔtHb (all of which would supplement the findings from the primary outcome measures). Any significant interactions within these RMANOVAs were further examined using *post hoc* comparisons employing a Tukey correction for multiple comparisons. Lastly, to determine the potential influence of condition order (CPT, HG, and CPT + HG), a RMANOVA was used to compare the resting MAP, SBP, DBP, and HR values across trials. All statistical analyses were conducted using jamovi (2.6.13.0) and the R statistical package, and significance was accepted as *p* < 0.050.

## Results

### Participant demographics

As noted previously, a total of fifty-five participants were included in this analysis, twenty-one of whom were classified as hyperreactors to the CPT (change in MAP, SBP, or DBP ≥15 mmHg). Participant demographics can be found in Table 1, which also includes a comparison of resting data between the NPR and HPR groups. Of note, individuals in the HPR group did not demonstrate any significant differences in resting SBP, DBP, HR, cBRS_all_, cBRS_up_, cBRS_down_, or forearm reactive hyperemia responses (TTP) compared to the NPR group (all *p* ≥ 0.205). Likewise, Table 2 includes baseline data compared between males and females and across racial groups. Lastly, neither MAP, SBP, DBP, or HR were significantly different at baseline across conditions (all *p* ≥ 0.124).

### Cold pressor test responses

**FIGURE 2:**
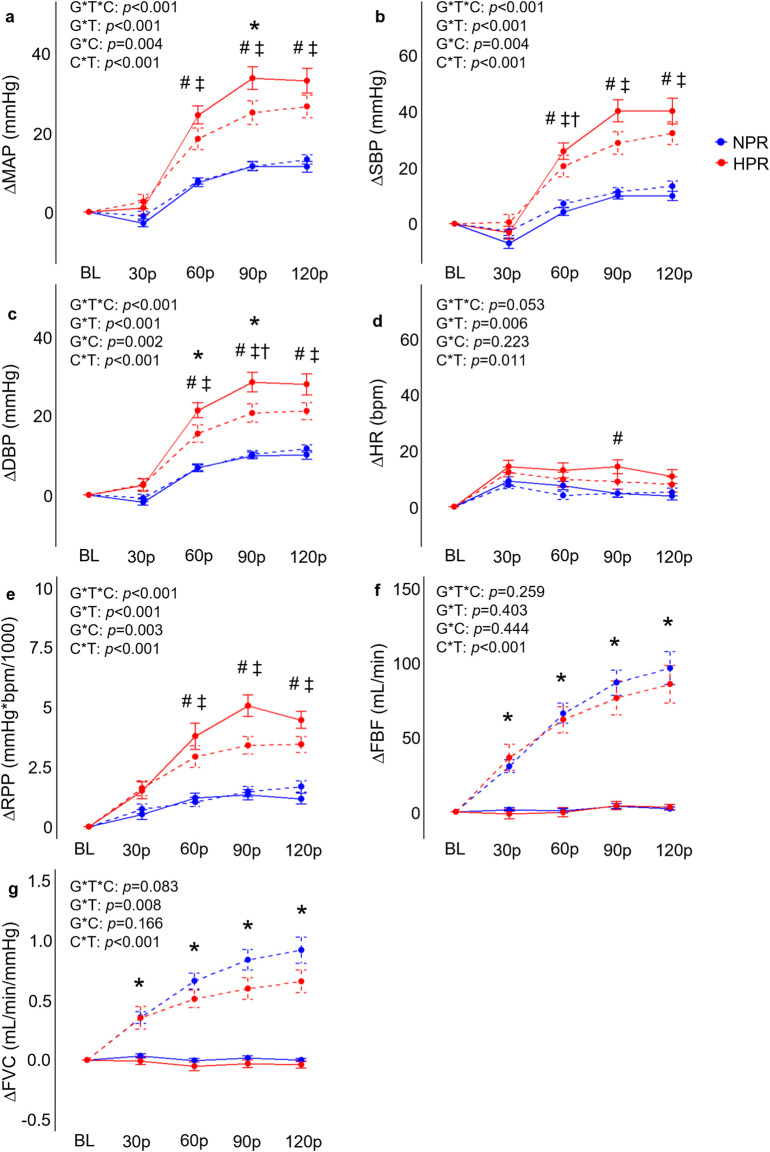
Comparisons of the relative changes in mean arterial pressure (ΔMAP; **(A)**, systolic blood pressure (ΔSBP; **(B)**, diastolic blood pressure (ΔDBP; **(C)**, heart rate (ΔHR, **(D)**, rate-pressure-product (ΔRPP; **(E)**, forearm blood flow (ΔFBF; **(F)**, and forearm vascular conductance (FVC; **(G)** compared between hyperreactive (HPR) and normoreactive (NPR) groups across time and between conditions (cold-pressor test only [CPT] vs CPT with combined handgrip exercise [CPT + HG]). All indices of blood pressure, ΔHR, and ΔRPP significantly increased more in the HPR group during the CPT trial compared to the NPR group, whereas ΔFBF and ΔFVC responses to the CPT were not different between groups. In contrast, the CPT + HG condition elicited significantly higher ΔFBF and ΔFVC in both groups, whereas the CPT + HG condition attenuated blood pressure responses compared to CPT alone in the HPR group. G*T*C, group by time by condition interaction; G*T, group by time interaction; G*C, group by condition interaction; C*T, condition by time interaction; #, significant difference between group for CPT trial only; *, significant difference between the CPT and CPT + HG conditions independent of group; †, significant difference between the CPT and CPT + HG conditions for the HPR group only; ‡, significant difference between groups for the CPT + HG condition. Timepoints include baseline (BL), followed by 30-s increments throughout each trial (30p, 60p, 90p, and 120p). Significance accepted at *p* < 0.050.

**FIGURE 3:**
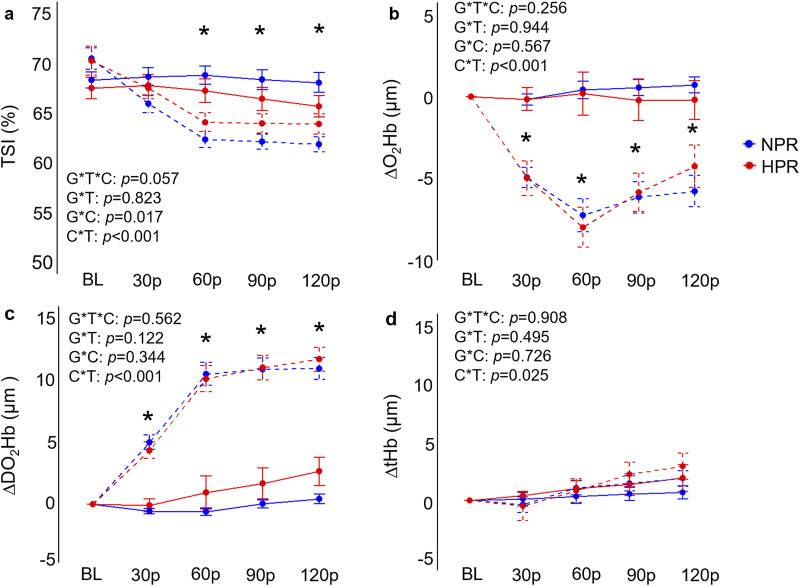
Comparisons of the relative changes in near-infrared spectroscopy derived tissue saturation index (TSI; **(A)**, oxygenated hemoglobin concentration (ΔO_2_Hb; **(B)**, deoxygenated hemoglobin concentration (ΔDO_2_Hb; **(C)**, and total hemoglobin concentration **(D)** compared between hyperreactive (HPR) and normoreactive (NPR) groups across time and between conditions (cold-pressor test only [CPT] vs CPT with combined handgrip exercise [CPT + HG]). TSI and ΔO_2_Hb both significantly decreased in the CPT + HG trial compared to the CPT trial independent of group, whereas DOH_2_Hb significantly increased. G*T*C, group by time by condition interaction; G*T, group by time interaction; G*C, group by condition interaction; C*T, condition by time interaction; *, significant difference between the CPT and CPT + HG conditions independent of group. Timepoints include baseline (BL), followed by 30-s increments throughout each trial (30p, 60p, 90p, and 120p). Significance accepted at *p* < 0.050.

As expected, individuals in the HPR group demonstrated significantly exaggerated ΔMAP (*f* = 37.9, *p* < 0.001), ΔSBP (*f* = 32.8, *p* < 0.001), ΔDBP (*f* = 35.6, *p* < 0.001), ΔHR (*f* = 4.57, *p* = 0.001) and ΔRPP (*f* = 27.1, *p* < 0.001) responses to the CPT compared to the NPR group (Figure 2), with differences generally reaching a peak at the 90p timepoint between groups (*p* ≤ 0.020). In contrast, no significant group by time interactions were observed for ΔFBF (*f* = 0.43, *p* = 0.790) or ΔFVC responses to the CPT (*f* = 0.70, *p* = 0.591) when the CPT trial was interrogated independently (Figure 2). A significant group by time interaction was observed for ΔDO_2_Hb (*f* = 2.79, *p* = 0.028) when the CPT condition was interrogated independently, which was explained by an increase in ΔDO_2_Hb from 30p to 120p in the HPR group (mean difference = 2.77 ± 0.60 µM, *p* < 0.001), which was not observed in the NPR group (all *p* ≥ 0.056; Figure 3). No other group by time interactions were observed for TSI, ΔO_2_Hb, or ΔtHb during the CPT trial (all *p* ≥ 0.094).

### Handgrip responses

**FIGURE 4:**
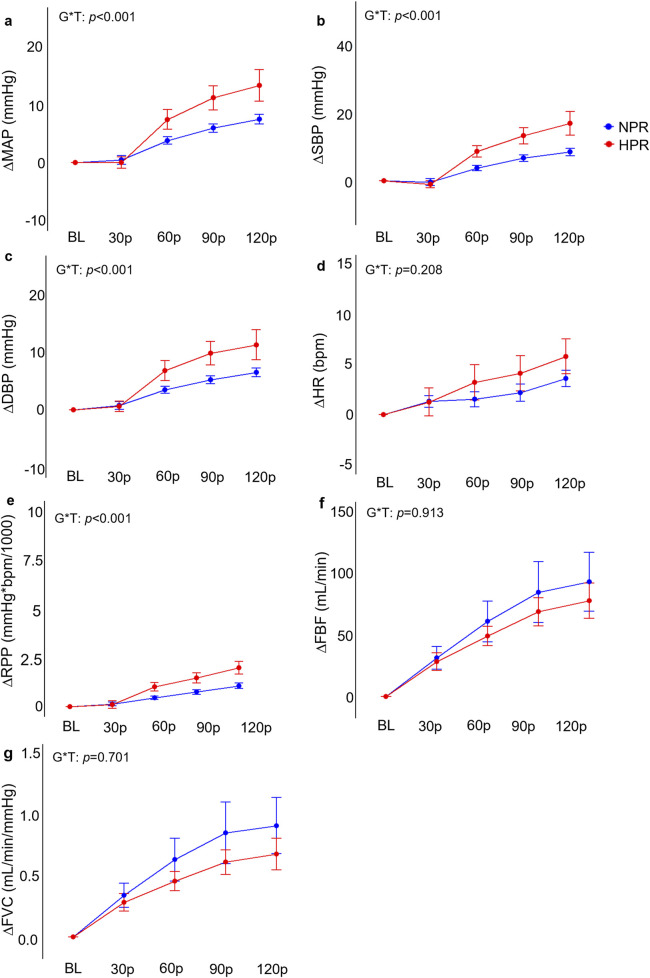
Comparisons of the relative changes in mean arterial pressure (ΔMAP; **(A)**, systolic blood pressure (ΔSBP; **(B)**, diastolic blood pressure (ΔDBP; **(C)**, heart rate (ΔHR, **(D)**, rate-pressure-product (ΔRPP; **(E)**, forearm blood flow (ΔFBF; **(F)**, and forearm vascular conductance (FVC; **(G)** compared between hyperreactive (HPR) and normoreactive (NPR) groups across 2 minutes of handgrip exercise. Significant group by time interactions were observed all blood pressure indices and ΔRPP, but not for ΔHR, ΔFBF, or ΔFVC responses. G*T, group by time interaction. Timepoints include baseline (BL), followed by 30-s increments throughout each trial (30p, 60p, 90p, and 120p). Significance accepted at *p* < 0.050.

**FIGURE 5:**
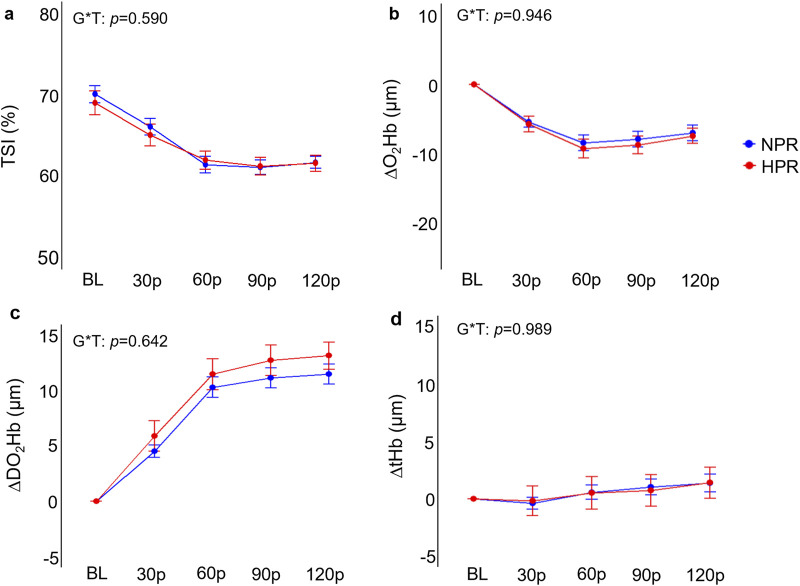
Comparisons of the relative changes in near-infrared spectroscopy derived tissue saturation index (TSI; **(A)**, oxygenated hemoglobin concentration (ΔO_2_Hb; **(B)**, deoxygenated hemoglobin concentration (ΔDO_2_Hb; **(C)**, and total hemoglobin concentration **(D)** compared between hyperreactive (HPR) and normoreactive (NPR) groups across 2 min of handgrip exercise. G*T, group by time interaction. Timepoints include baseline (BL), followed by 30-s increments throughout each trial (30p, 60p, 90p, and 120p). Significance accepted at *p* < 0.050.

Individuals in the HPR group demonstrated significantly exaggerated ΔMAP (*f* = 6.40, *p* < 0.001), ΔSBP (*f* = 7.17, *p* < 0.001), DBP (*f* = 5.01, *p* < 0.001), and ΔRPP (*f* = 8.18, *p* < 0.001) responses to HG compared to the NPR group (Figure 4). When examined further using *post hoc* analyses, these change scores failed to demonstrate statistically significant differences between groups at the 30p, 60p, 90p, and 120p timepoints. In contrast, neither ΔHR (*f* = 1.48, *p* = 0.208), ΔFBF (*f* = 0.24, *p* = 0.913), or ΔFVC (*f* = 0.54, *p* = 0.701) demonstrated any significant group by time interactions for the HG trial, nor did TSI (*f* = 0.70, *p* = 0.590), ΔO_2_Hb (*f* = 0.19, *p* = 0.946), ΔDO_2_Hb (*f* = 0.66, *p* = 0.624), or ΔtHb (*f* = 1.48, *p* = 0.989; Figure 5).

### Combined effects of cold pressor activation and exercise induced hyperemia

When examining whether CPT and HG responses interacted differently between groups, results demonstrated significant group by condition by time interactions for ΔMAP (*f* = 5.69, *p* < 0.001), ΔSBP (*f* = 5.16, *p* < 0.001), ΔDBP (*f* = 6.90, *p* < 0.001), ΔRPP (*f* = 6.28, *p* < 0.001), but not for ΔFBF (*f* = 1.33, *p* = 0.259), ΔFVC (*f* = 2.10, *p* = 0.083), ΔHR (*f* = 2.38, *p* = 0.053), TSI (*f* = 2.33, *p* = 0.057), ΔO_2_Hb (*f* = 1.34, *p* = 0.256), ΔDO_2_Hb (*f* = 0.75, *p* = 0.562), or ΔtHb (*f* = 0.25, *p* = 0.908). Post-hoc analyses indicated that the HPR group demonstrated significantly elevated ΔMAP, ΔSBP, ΔDBP, and ΔRPP responses across 60p, 90p, and 120p in the CPT + HG trial compared to individuals in the NPR group (all *p* ≤ 0.010; Figure 2). Moreover, when the responses to the CPT and CPT + HG conditions were compared within each group independently, results indicated significant condition by time interactions for ΔMAP (*f* = 6.95, *p* < 0.001), ΔSBP (*f* = 6.44, *p* < 0.001), and ΔDBP (*f* = 6.82, *p* < 0.001) in the HPR group, none of which were observed in the NPR group (all *p* ≥ 0.164). These interactions were explained by significantly lower ΔSBP responses during CPT + HG compared to CPT at 90p in the HPR group (mean difference = 11.4 ± 3.1 mmHg, *p* = 0.048), as well as significantly lower ΔDBP responses at 60p (mean difference = 5.9 ± 1.6 mmHg, *p* = 0.032; Figure 2). Significant condition by time interactions were observed for all variables (all *p* ≤ 0.025).

## Discussion

This study tested the primary hypothesis that hyperreactive blood pressure responses to the CPT would be accompanied by similar increases in peripheral vasoconstriction. Based on the observation that neither ΔFBF and ΔFVC were significantly different between the HPR and NPR groups, despite significantly exaggerated blood pressure, ΔHR, and ΔRPP responses in the HPR group, our findings do not support this hypothesis. Instead, these findings suggest that hyperreactive responses to the CPT occur independent of peripheral vasoconstriction responses, and also indicate that hyperreactive blood pressure responses to the CPT are attenuated with concomitant HG exercise. Possible explanations for these findings are discussed in more detail in the following sections, along with their implications.

### Peripheral vasoconstriction is not exaggerated in CPT hyperreactors

The primary finding that neither FBF nor FVC responses were significantly different during the CPT in individuals classified as hyperreactors, despite significantly exaggerated blood pressure responses, is surprising and contrary to the study hypothesis. Consistent with prior studies (Moynes et al., 2013; Miller et al., 2019), the relative changes in FBF, and to a greater extent FVC, were used as indices of peripheral vasoconstrictor responses during the CPT. The hypothesis that peripheral vasoconstriction would be exaggerated in the HPR group is based on prior studies demonstrating significant increases in MSNA (Victor et al., 1987; Yamamoto et al., 1992), total peripheral resistance (Ifuku et al., 2007), and pulse wave velocity [PWV; (Moriyama and Ifuku, 2010)] during the CPT, as well as the observation that individuals classified as hyperreactors have also been reported to demonstrate exaggerated increases in pulse-wave augmentation index normalized to a HR of 75 bpm (a common index of arterial stiffness and wave reflection) compared to controls (Moriyama and Ifuku, 2010). However, as noted previously, our findings refuted this hypothesis. Not only were the ΔFBF and ΔFVC responses to the CPT not different between groups (Figure 2), but there were also no significant differences in the ΔtHb responses to the CPT (Figure 3). While this may seem to disagree with prior evidence (particularly regarding changes in pulse wave characteristics), it is also important to recognize that, to the authors’ knowledge, there are no studies that have directly compared MSNA or peripheral vasoconstrictor responses between hyperreactors and normoreactors to the CPT. Therefore, while pulse wave characteristics may provide a valuable index of vascular tone, there is no direct evidence of augmented peripheral vasoconstriction in hyperreactive responses to the CPT. The data presented in this study would support the notion that peripheral vasoconstriction does not contribute to these hyperreactive responses, and instead points to augmented HR responses as the primary driving factor. However, it is also important to recognize that this study is observational in nature (no experimental manipulation of vasoconstrictor responses during the CPT), and therefore cannot directly infer a causal relationship. Considering prior evidence that cardiac contractility (estimated via the carotid pressure wave) is not exaggerated in hyperreactors (Moriyama and Ifuku, 2007), it may be possible that these hyperreactive responses are explained by CPT mediated withdrawal of cardiac parasympathetic activity, as also proposed by Ifuku (2015).

In addition to these observations, it was also notable that DO_2_Hb significantly increased towards the end of the CPT trial in the HPR group, but not the NPR group (Figure 3). It has been demonstrated that cold stress induces pronounced increases in central venous pressure (Wilson et al., 2007), and prior studies have demonstrated that cold exposure significantly increases skin sympathetic outflow in individuals with hypertension (Greaney et al., 2017), ultimately leading to reductions in cutaneous perfusion. In the present study, significant increases in central venous pressure may be sufficient to slow venous return, leading to the accumulation of DO_2_Hb in the NIRS sampling region in the HPR group. However, this remains speculative, and additional work is needed to expand on these observations.

### Blood pressure responses during CPT and CPT + HG

Given that we did not observe any significant exaggeration of peripheral vasoconstriction in the HPR group, we would not expect to see any attenuating influence of CPT responses on HG-induced hyperemia in the HPR group, as the secondary hypothesis proposed. This notion is supported by our findings, as we did not report any significant three-way interactions for ΔFBF or ΔFVC. However, we did observe an apparent inhibitory influence of HG on CPT responses, characterized by a decrease in blood pressure responses from the CPT to the CPT + HG trials in the HPR group, whereas neither ΔSBP, ΔDBP, or ΔMAP were different between the CPT to CPT + HG trials in the NPR group (Figure 2). Even more perplexing is the observation of significant group by time interactions for the HG trials, explained by greater responses in the HPR group. Therefore, it seems that the combination of these two hypertensive stimuli appears to be inhibitory in individuals with a hyperreactive response to the CPT. It is also important to recognize that these findings contrast with the findings of Moriyama and Ifuku (2010), who report no exaggeration of central cardiovascular reactivity during HG exercise in hyperreactors, and with the findings of Geleris et al. (2004), who report an additive effect of CPT and isometric HG exercise on blood pressure responses. However, the study by Geleris et al. (2004) did not differentiate between NPR and HPR groups, and both studies employed isometric HG exercise protocols, in contrast to the rhythmic HG exercise protocols employed in the present study. The purpose for employing a low-intensity (25% MVC) rhythmic HG protocol in this study was to maximize forearm blood flow, which could have been partially occluded with higher intensity (≥30% MVC) isometric handgrip force (Byström and Kilbom, 1990). These factors likely contribute, in part, to the contrasting findings between studies. Nevertheless, the inhibitory effect of combined CPT + HG observed in the present study remains of interest. One plausible explanation for this effect could be a centrally mediated inhibitory effect, whereby descending motor signals (and thus, central command) inhibit the relay of the sensory reflex contributing to exaggerated CPT responses. This notion is supported by prior evidence that stimulation of the motor cortex inhibits nociceptive feedback and increases the pain threshold in animal models (Pagano et al., 2011; Lopes et al., 2019). Considering that pain has also been shown to be greater during the CPT in individuals demonstrating a hyperreactive response (Peckerman et al., 1991; Moriyama and Ifuku, 2007), a reduction in nociceptive feedback during the combined condition may explain this attenuating effect.

### Implications and future directions

The understanding that hyperreactive responses to the CPT are not accompanied by exaggerated sympathetic vasoconstriction has a few important implications for future research. First, these findings indicate that, while the CPT may be useful for detecting increased risk of future cardiovascular disease and hypertension (Wood et al., 1984; Kasagi et al., 1995; Han et al., 2022), this test may not be a suitable measure for detecting exaggerated vasoconstrictor responses. Instead, future research may consider similar analyses using other vasoconstrictor stimuli, such as graded limb suction (Lott et al., 2009). Second, the findings of this study add to the understanding of the mechanisms contributing to exaggerated blood pressure responses to standard sympathetic stimuli. While causality cannot be inferred in the present study, these findings would support the notion that peripheral vasoconstriction is unlikely to explain exaggerated CPT responses, which could be confirmed with further experimental research. Elucidating the factors that contribute to these exaggerated blood pressure responses, which are known to be associated with increased disease burden (Wood et al., 1984; Kasagi et al., 1995; Han et al., 2022), may eventually lead to the identification of therapeutic targets for the prevention of cardiovascular disease.

In addition to the primary findings regarding CPT responses, the finding that combined CPT + HG exerts an inhibitory influence on the hyperreactive blood pressure response to the CPT alone is also potentially beneficial to individuals at an elevated risk of hypertension. If acute muscular contractions can mitigate exaggerated blood pressure responses to nociceptive sensory inputs, this may provide an avenue by which acutely exaggerated blood pressure responses can be controlled. However, further research is needed to determine if these findings extend to other acute stimuli, such as whole-body cold exposure or pain responses.

### Limitations

Consistent with any study, there are certain aspects of this investigation that should be taken into consideration when interpreting the findings. First, there are several physiological variables that were not collected in this investigation, including direct assessments of MSNA, subjective pain responses, cardiac output, pulmonary ventilation (i.e., L/min), and total peripheral resistance. While prior studies have consistently demonstrated robust increases in MSNA during the CPT (Victor et al., 1987; Yamamoto et al., 1992), due to the lack of MSNA data in the present study, no inferences can be made regarding sympathetic reactivity or sympathetic vascular transduction between the HPR and NPR groups. Likewise, the lack of subjective pain responses also limits the ability to determine if the attenuated blood pressure responses in the CPT + HG condition in the HPR group was partially explained by reduced pain responses. Other factors, such as genetic and psychological factors related to cold perception, may also provide further insight into potential differences between the responses observed in the HPR vs NPR groups. While these factors may be beyond the scope of the present study, these would be important considerations for future studies aimed at identifying the mechanisms contributing to exaggerated sympathetic reactivity. It is also important to recognize that the order of experiments was not randomized nor counterbalanced in this study, which assured that CPT responses (used to test the primary hypothesis) were unaffected by prior exercise. However, it is also worth noting that neither MAP, SBP, DBP, or HR were significantly different between the baseline timepoints of each condition (all *p* ≥ 0.124), suggesting that the 10-min rest period between trials allowed a return to baseline levels. Likewise, it is important to acknowledge that limb selection for a CPT has been shown to influence the magnitude of the sympathetic response, with hand-immersion eliciting greater increases in MSNA and blood pressure compared to foot-immersion (Coovadia et al., 2024). In the present study, foot-immersion was selected to allow both hands to be available for HG exercise and beat-by-beat blood pressure assessment, respectively. While the foot-immersion technique employed in the present study still elicited robust differences in central hemodynamic responses between groups, future studies may consider extending these findings by comparing the vasoconstrictor responses to hand-immersion. Lastly, water temperature was not directly controlled during the CPT trials in this study, and instead, water temperature was recorded during a subset of CPT trials (*n* = 3). Despite the lack of water temperature control, water temperature was consistently recorded between 3° and 4°C throughout each recorded trial.

### Perspectives and significance

In conclusion, this study found that peripheral vasoconstriction, reported as the relative changes in FBF and FVC, was not different during the CPT in individuals classified as hyperreactors. Furthermore, this study also found evidence that combined CPT + HG exerts an attenuating effect on blood pressure responses to the CPT in individuals classified as hyperreactors, suggesting that descending motor drive (and thus, central command) may counteract afferent mediated hyperresponsiveness to the CPT. These data support the notion that peripheral vasoconstriction likely does not explain hyperreactive blood pressure responses to the CPT, and support future experimental work aimed at identifying the mechanistic factors contributing to this response.

## Data Availability

The raw data supporting the conclusions of this article will be made available by the authors, without undue reservation.
